# How to Recover from a Brain Disease: Is Addiction a Disease, or Is there a Disease-like Stage in Addiction?

**DOI:** 10.1007/s12152-017-9312-0

**Published:** 2017-03-13

**Authors:** Anke Snoek

**Affiliations:** 1 Faculty of Health, Medicine & Life Sciences, Maastricht University, Peter Debyeplein 1, 6229 HA Maastricht, The Netherlands; 2 Faculty of Arts, Philosophy Department, Macquarie University, Sydney, NSW 2109 Australia

**Keywords:** Addiction, BDMA, Recovery, Qualitative research, Lewis, Agency, Compulsion, Duress

## Abstract

People struggling with addiction are neither powerless over their addiction, nor are they fully in control. Lewis vigorously objects to the brain disease model of addiction (BDMA), because it makes people lose belief in their self-efficacy, and hence hinders their recovery. Although he acknowledges that there is a compulsive state in addiction, he objects to the claim that this compulsion is carved in stone. Lewis argues that the BDMA underestimates the agency of addicted people, and hence hinder their recovery. Lewis’s work offers us a very much to be welcomed neurobiology of recovery. It offers addicted people a hopeful and respectful narrative for their recovery that treats them as agents rather than as damaged brains. However, I argue that overestimating people’s agency can also result in people losing belief in their self-efficacy. Lewis’s strong focus on the agency of addicted people might not match their experiences of struggle, hence reinforcing their feelings of guilt when they fail to control their use. I propose to replace the notion of addiction as a disease with a notion of a disease-like stage in addiction. I call this stage the duress stage in addiction, in which the addictive behaviour is largely impervious to the agent’s values and to available techniques of self-control. However, the agent can overcome this stage by developing new techniques of self-control, by building on their self-concept and belief in self-efficacy, by changing their environments and habits, and by engaging in projects that are meaningful to the agent.

## How to Recover from a ‘Brain Disease’?

Lewis provocatively subtitles his book: ‘Why addiction is not a disease’ [1]. He especially seems to object to the brain disease model of addiction (BDMA). The BDMA consists of three parts. The first part is a technical description of how repeated substance use influences brain chemistry. For example, there is how the dopamine released in substance use influences attention and makes us highly sensitive to substance-related cues. The second part consists of normative claims (closely or less closely) based on these described mechanisms. We have to, for example, translate these mechanisms into human behaviour, into a more normative and less technical language. Dopamine release during substance use provides an extreme reward and this leads to continued use, and eventually compulsive use. However, the BDMA also makes a normative overarching conclusion on their findings: that addiction is a chronic, relapsing brain disease. The third part, which is closely related to the normative claims, consists of social goals the BDMA has set. For example: providing an alternative for the moral model of addiction, reducing stigma, improving treatment.

Lewis claims that the aim of his book is to critically examine the neuroscientific foundation of the disease model. However, the neuroscientific mechanisms Lewis describes as underlying addiction are largely in line with the general consensus in this field. He acknowledges that addiction involves harmful behaviour, and in some parts of his book he even seems to support the view that there is a compulsive stage in addiction.^1^ Lewis might change an accent here or there in describing the neuroscientific mechanisms of addiction but he largely seems to agree with the BDMA on the mechanisms described. His main objection is to how the neuroscientific mechanisms are translated into normative statements. Lewis’s main goal seems to be to show that the normative conclusions of the BDMA do not follow logically from the neuroscientific mechanisms described. The BDMA proponents present their model as a logical outcome of scientific research, but most of their conclusions are normative and partly inspired by their scientific research and partly by their social goals.

How we describe a phenomenon often depends on what we are interested in. A nice meal my grandmother made can be described in terms of cooking techniques or in terms of family traditions, depending on what I am mainly interested in. If we look at the historical setting in which the BDMA was developed, we see that it was developed to oppose simple choice models like the moral model. The moral model claims that addicted people are in control of their behaviour. The view on human agency this model holds is that to understand why people behave a certain way, we have to look at what reasons they have to behave that way. Addicted people find pleasure in use, or relief from pain, so they act in a self-controlled way. This view on addiction has led to stigmatisation of addicted people, and hinders people when they seek treatment. The BDMA tried to show that our behaviour is not only determined by reasons, but also by causes [2]. Repeated substance use changes our attention and our impulse control. Hence, addicted behaviour is largely caused by sensitisation to substance- related cues, and a dramatic reduction in cognitive control. The social goals of the BDMA were to provide an alternative to the moral model of addiction, to reduce stigma, and to stimulate people to seek treatment. Hence, they overemphasised the compulsive stage in addiction. In the most extreme version of the BDMA, these social goals led to a view on human agency of addicted persons determined by causes, rather than reasons. The BDMA tries to explain (against the moral model) why people at the height of their addiction find it so hard to control their use (because their behaviour seems to be determined more by cues than reasons).

Lewis however, has a different social goal in mind. His main interest seems to be to understand recovery (rather than explaining compulsion or involuntary behaviour in addiction as the BDMA does), and from this focus he approaches the complex phenomenon of addiction. His vigorous attack on the BDMA’s normative claims is motivated by a social goal: to improve the chances of recovery which he thinks the BDMA gets wrong; in fact he thinks the BDMA hinders recovery. This does not mean however that we should steer away from the neurobiology of addiction, as most people tend to do [1, p.24]. Lewis argues that based on the same neurological data we can draw different normative conclusions, namely that addiction is deep learning; a severe entrenched habit.

One can object that once we understand how addiction works neurologically, we know how to treat it, but as Lewis points out, although the BDMA had certain hopes for recovery (reducing sigma, offering better treatment through medicine that target brain mechanisms), so far it has failed to fulfil those hopes. Addiction cannot be diagnosed through brain scans, nor is it treated with medicines (although medication can support treatment). Brain scans of addicted people have very little prognostic value. This does not mean that neuroscience is not important, only that it isn’t telling the whole story.

Lewis is right, the BDMA is missing something important about recovery. It is not designed to describe recovery. Miller has already argued that though animal models are very useful for understanding how someone gets trapped in addiction, there is no adequate animal model of recovery.^2^ This is because animal models seem to miss something important about human agency [3, p. 63].^3^ Lewis is a neuroscientist, he understands the animal models, yet, he finds the strong focus on neuroscientific explanations of addiction unsatisfactory, probably because from his own experience with recovery from substance dependency he learned some important things about human agency. So what has Lewis learned about agency based on his own experiences with addiction?

## Lewis’s Neurobiology of Recovery and Agency

Crucial to Lewis’s description of the neurobiology of addiction and recovery is the relationship between the dorsolateral prefrontal cortex (PFC), or what he calls the bridge of the ship, and the striatum, or the motivational engine. When people start using substances, the use often has a positive effect on their lives: it helps them to relax better in social situations, or to perform better at work. Especially for people who struggle with unresolved childhood issues, issues they are sometimes even not aware of, substance use can be highly effective. In this stage, the dorsolateral PFC supports the use because it solves a problem people don’t know how to solve otherwise. What follows is a process of deep learning that is similar to other processes of learning: we form a habit fuelled by a desire. However, with substance use this process goes faster than with other habit formations. The habit becomes entrenched quickly. In this stage,

the dorsolateral PFC becomes partially disconnected from the striatum. The bridge of the ship loses its command over the motivational engine. (…) The reasons for this disconnection are complex and not fully understood. But suffice it to say that habits free themselves from higher-order controls because the striatum no longer sends out requests for prefrontal engagement’ [1, p.187].

Once this connection is distorted, ‘desire drives behaviour in small redundant circles, independent of insight, perspective, and higher-order goals’ [1, 205].

So far Lewis’s description corresponds largely with that of the neuroscience of addiction in general. However, where neuroscience stays silent about how this disconnection can be restored, Lewis takes the analysis a step further. Mostly people describe the role of the prefrontal cortex within a dual processing model: as cognitive control, as willpower. However, just trying to control our use will only hasten ego-depletion [4].^4^ Lewis however, argues that there is a different way in which the dorsolateral PFC can reconnect with the motivational engine.

As long as addiction is tolerated by the user, Lewis argues, nothing happens, but when addiction is no longer tolerated, people can actively try to reconnect with the bridge of the ship. The way to do this is not to focus on discontinuing use, but to imagine a valued future, and try to make it accessible. We can try to shift our perspective and attention rather than supress our urges [1, 158]. When we do this, we can reconnect the motivational engines with the bridge of the ship, and we can see the horizon again [1, 192].^5^ In order to achieve this, Lewis sees an important role for self-narrative and self-trust. ‘The addict’s life is lived in the tomb of the present, dead because it has lost its connection with the story from which it came’ [1, 205], Lewis argues. He cites for example a study on suicide amongst native Americans. When native American communities with a high suicide rate were compared to those with a low suicide rate, it was found that those more prone to suicide had no stories to tell about their lives. When asked about their goals, their past, their future, they had no narrative [5]. Lewis stresses the importance of making sense of one’s own life, giving it meaning and purpose.

It appears that Natalie and Brian began to outgrow their addictions when they were able to reflect on their lives, connect their past to their present conundrum, and imagine a future very different from the present. (…) As a result, they no longer had to fight their impulses with the same exhausting effort hour by hour and day by day. This greatly reduced ego fatigue, which was the key to changing momentary behaviour and learning to rely on top-down control. (…) They could detect a shift in the course of their own personality development: the emergence of new mental habits, new habit of behaviour, and a different sense of who they were as people. [1, p.201-2]

In the case studies he presents, redefining one’s identity and narrative plays an important role in recovery. The people in his case studies examine which problems in their lives they believe they could only solve with substance use, and how they can address these problems differently, mostly by redefining their identity and their self-narrative.

Brian had to fashion a story, a narrative, to explain himself to himself. (…) Instead of seeing himself as a classic victim of failed relationships, he began to imagine that he was the kind of person who could fix them, for others as well as himself. (…) Pieces of a puzzle seemed to come together with insight, luck, and time. (…) Brian emerged as someone who could bring order to chaos, gather the lost ones and help them find their way home. This image of himself has grown from a daydream to a burning commitment over the years since then. [8, p.90-91]

So what is the BDMA missing about agency? Traditionally there are two dominant views on human agency in addiction. The first view states that to understand why people behave a certain way, we have to look at their reasons: what’s in it for them? These are the simple choice models of addiction. The second view on human agency states that human behaviour is not only motivated by reasons, but also by neurological, genetic or social *causes* [2, p.471]. The BDMA focuses mostly on these causes of behaviour. Lewis represent a third, less known school that looks at the role of identity or self in motivating behaviour [2, 6, 7]. We can exercise self-control by exercising cognitive control, but we can also exercise self-control by changing our self-concept. As Lewis states in one of his case-studies: ‘Natalie had to find a self before she could find self-control’ [1, p.67]. We estimate what others are likely to do, based on what we know of their character and history: will my friend be on time, or will she be late as usual? In the same way we make estimations about ourselves based on our self-concept. Our self-concept has a role in determining what kinds of choices are presented to us internally, as part of our character. Our self-concept determines our motivation [8].

This view of Lewis on the importance of self-concept and self-narrative in recovery is supported by research. Biernacki for example wondered why some people spontaneously recovered from heroin addiction. He interviewed 101 ex-heroin users who quit their use without professional intervention, and found that fundamental to their recovery was an identity change [9]. Many qualitative studies on recovery have since supported these findings [10–14]. There are also a few case studies on narrative treatment that show promising results [15–17]. The BDMA not only largely ignores identity as a source of human agency, Lewis fears that the message the BDMA sends will have a negative effect on people’s self-concepts, and thus negatively influence their recovery.

## The Merits and Risks of Lewis’s Model

There are many merits to Lewis’s model of recovery. One merit is that he connects what happens on an existential level to what happens on a neurobiological level. He explains how in changing our narrative, we can reconnect our motivational engine with the bridge of the ship from where we can see the horizon again. He offers a neurobiological and existential account of recovery.

Maybe the largest merit of Lewis’s model is that he offers a positive narrative on recovery. Most available narratives on recovery represent substance users as damaged goods. The current neuroscientific models especially are dominated by messages of long-term, even irreparable damage to the brain. Most recovery narratives are redemption stories in which the addicted person realises he or she was wrong, and in which an effort was made to try to restore some normality, while admitting that their substance use may have damaged them irreparably [18]. Lewis’s model provides us with a much needed, positive narrative on recovery. Lewis offers an alternative to the neuroscientific narrative of irreparable damage to the brain. He cites a study on how grey matter is restored after someone overcomes addiction [1, p. 137]. After six months to a year of abstinence, grey matter returns to its normal baseline level. But as abstinence continues, the grey matter volumes keep increasing, beyond the normal baseline levels of the controls that have never been addicted. What is also remarkable is that the regions in which the growth occurs do not exactly correspond with the regions that were depleted [19]. In overcoming one’s addiction, people don’t return to their old state, but they have learned new things, they have grown. Lewis objects to the word ‘recovery’ because it suggests that something is repaired, and it suggests that a person has returned to a status quo, but in his developmental perspective, a person has grown.

Lewis’s model is especially positive for people with comorbid mental illness. This group is often presented as having the worst prospects on recovery. They are less likely to mature out of their addiction [20, 21], they tend to have worse treatment outcomes. The addiction makes it harder to treat the mental illness, while the mental illness makes it harder to treat the addiction, and on top of that, the substance aggravates the mental health problems [22]. However, by sketching a broader picture of a developmental trajectory rather than focussing narrowly on mental illness, Lewis gives a more hopeful message for treatment. ‘Addiction need be no more than a stage in the development of the self. And that often seems to be exactly what it is.’ [1, p.215]. Addiction is not in itself the problem, addiction is just an extension of ‘a developmental pattern already set in motion’ [1, p. 36], and can be overcome by overcoming problems of the self.

Lewis’s intuition that the normative message of the disease model is detrimental to recovery seems also to be backed up by research. One study demonstrated that people who support the disease model are less likely to recover [23].^6^ However, this is the point I would like to explore further. If Lewis’s social goal is to improve recovery, does he help people best by explicitly rejecting the disease model? I see the benefit of promoting agency for recovery, a strategy that Pickard has also outlined [24], however, I wonder how this agency can best be promoted. Lewis is puzzled by the response he got from one of his readers:

But what really moves me is the addicts who get in touch and say, ‘Don’t take this away from me. If you take away the disease label, then basically I won’t be able to get better, if you don’t let me understand myself as having a disease.’ It’s a very strange argument, to have to think of yourself as having a disease because that’s the only way you can live with yourself and deal with the addiction. And then I feel badly, because I don’t want to harm these people or take away something that they need conceptually or motivationally. (…) They feel that if it is a disease, they don’t have to feel that burden or shame, because it’s not their fault. It’s hard to pull the rug out from under that without causing some upset. [25]

I will explore this objection to Lewis’s account in two ways. Firstly I will argue that Lewis can prevent these kinds of objections by specifying which normative claims of the disease model he mainly objects to, instead of broadly stating that addiction is not a disease. Secondly I will argue that the Lewis model of addiction and recovery would have been stronger if he did not engage with the disease discussion. Although Lewis explicitly objects to the dichotomy that addiction is either a disease or a choice, by so strongly objecting the disease model, he risks staying trapped in the dichotomy, and his model being mistaken for a simple choice model. I will argue that both a disease model and a simple choice model of addiction are detrimental to recovery. I will suggest a third way in which we can look at agency in addiction inspired by research on belief in self-efficacy.

## What Does It Mean to Call Addiction a Disease?

Whether it is beneficial to recovery or not to adopt the disease model all depends on which normative claims of the disease model we adopt. Adopting a disease view on addiction does not necessarily mean that one adopts an overly pessimistic view on their agency. In my qualitative follow-up research among 69 alcohol and opioid dependent people, I asked them several questions on how they perceived their agency, and one of my questions was whether they thought addiction was a disease.^7^ In the first year the majority (68%) stated that addiction was indeed a disease. Only 13% of the people explicitly stated that addiction was not a disease and 19% were ambivalent. The second year we decided to slightly rephrase the question to: ‘Some people say that addiction is a (brain) disease, others claim that it is a choice. What do you think?’ Suddenly respondents expressed much more ambivalence about the disease concept: 55% said that it was both a choice and a disease, 32% stated it was a disease, 10% claimed it was a choice, and 3% didn’t know.

There are several explanations for this shift. The first year I recruited about half of my sample in a detox facility (the other half of the sample was recruited from a maintenance treatment program). So half of the respondents might have been at a stage in which they felt they had very little control over their substance use. This finding could mean that people seek treatment when they think their addiction is a disease.

However, another explanation is that addicted people who endorsed the disease model didn’t think that they had any choice left. So when we slightly rephrased the question, people expressed this view more easily. So what do addicted people mean when they say addiction is a disease? Which aspects of the disease model do they find beneficial?

The disease label emphasised for them the involuntariness of their condition.

If a disease is something you don’t want that you’ve got and that ruins your life, you know it is in a way… (R67)

However, the type of disease emphasised by study participants was interesting. It was remarkable that although in the first round of interviews many people supported the disease label, only three people saw it as a *brain* disease. The most common answer was that addiction is a hereditable disease, many respondents had parents or siblings who also struggled with addiction.

The disease model was also used to describe why they were different from other people, why they found it hard to just drink a limited amount, or just binge one day, like their friends.

Another reason why people supported the disease model was because they found it very hard to overcome their addiction. As a response to their loved ones, who often advocated a ‘just say no’-technique, the disease label fitted their experiences better:

I’m not too sure. It’s controversial. Some people ... like my parents don’t think that, they just think ‘oh get over it and stop’ but I think I can’t, but a lot of people do think it’s a disease but I don’t know. I don’t know. I’m not too sure. (R45)

my mum and dad thinks that it’s a choice you know so that’s why they’re pushing me away and thinking you know I’m choosing drugs over my son and my family when I’ve had a good life and I have you know I haven’t really got an excuse to use. (R57C)

Although most people are ambivalent about whether or not they have a disease, the disease label offers them protection from the simple choice models that their loved ones sometimes support. So when my respondents claim that addiction is a disease, they do not claim that they have no choice left due to how addiction changed their brains, they claim that they did not choose to become addicted, and that their condition is not easy to overcome. If Lewis had specified which normative claims of the disease model he objects to, maybe most addicted people who support the disease label for strategic reasons would have found that their view does not differ that much from Lewis’s.

When Lewis objects to the disease model he mostly seems to object to the normative claim that addiction is caused by something outside the agent, a dysfunction in the brain, and that the solution also lies external to the agent: in changing the brain through medicines. Instead of saying addiction is not a disease, Lewis should say: addiction is not a chronic, relapsing brain disease that can only be cured by an outsider with medicine. Lewis does not object to the idea that addictive behaviour can be compulsive; he objects to the idea that compulsion is carved in stone. It is especially this normative claim of the disease model that Lewis objects to because he thinks it will hinder recovery. Lewis thinks that in restoring one’s sense of self-efficacy, people can make important steps in recovery. He is afraid that the BDMA results in people underestimating their self-efficacy. Next I will argue that there is also a risk in overestimating one’s belief in self-efficacy, and that this is also detrimental to recovery. If Lewis had steered away from the whole dichotomy between choice and disease, his model would have been more nuanced.

## Addiction and Belief in Self-Efficacy

For people to try to change their situation, they have to believe that they are able to change it. They have to believe in their self-efficacy. Lewis is afraid that the BDMA hinders this belief in self-efficacy. However, not only underestimating our belief in self-efficacy can be detrimental to our recovery; overestimating it can as well. Belief in self-efficacy is often seen as a static condition that an individual can choose to have [26, 27]. As Bandura shows, however, belief in self-efficacy can be constructed or undermined through certain experiences [28]. For example performances: when we experience success it enforces our belief in self-efficacy, while failure diminishes it. So there is a risk that when we spark addicted people’s belief in self-efficacy, but they subsequently fail to control their use, they feel even more demoralised than before. A sense of shame and guilt on top of that will further fuel the addiction [29]. It is interesting that relapse hardly plays a role in Lewis’s case studies. As Huges argues: ‘The most important aspect to smoking cessation is maintaining the motivation to make multiple attempts’ [30]. People need several attempts to overcome their addiction because their agency needs to be restored on several levels. They simultaneously need to target the causes, reasons, and self-concepts that motivate their addictive behaviour. They will need a range of strategies to overcome their addiction [31], and this will take time. So when we tell people that they can overcome their addiction, we also need to tell them that it will be hard.

When I plan to climb the Mount Everest, I first need to believe that I can do it. It helps when people support me and tell me that they believe I can do it. But it is equally important that I am prepared for the hardship I will encounter and know some strategies for overcoming the hardship. We do want to appeal to people’s sense of agency, and motivate them to use what is left of it, however, we want to acknowledge as well that their agency is limited. In order to restore self-efficacy, we need to simultaneously point out that people are able to overcome their addiction and that this will be hard. Although most theories acknowledge both sides, there seems to be a tendency to focus on either control or control reducing factors. Lewis does this as well. If he would have just titled his book ‘the neurobiology of recovery’, it would have been a much stronger account. Although Lewis’s model is quite elaborate on why it is so hard to overcome addiction, by stating so strongly that addiction is not a disease, his model can be easily mistaken for a simple choice model. By so vigorously objecting to the disease model of addiction he distracts from what the main focus should be: giving people a balanced view on how their agency is diminished in addiction, and how they can overcome it.

As Berridge argued, overcoming addiction requires a special act of personal agency [32]. Lewis made a valuable attempt to show what this act of agency could look like, however, there is still quite a lot of work to do in this regard. Lewis might be too optimistic about the possibilities of narrative agency and identity change. Lewis himself already outlines how hard it is to adopt a narrative approach in addiction because in addiction we lose a linear sense of time, as we are trapped in the now-appeal substances reinforce. In addition, the literature on the role of identity in the recovery from addiction, show that for some people it is easier than for others to overcome addiction through the motivational force of their identity [8, 35, 36]. In order to change our story and self-concept, we need to have identity materials. Some people might have more identity materials than others, because their addiction use started later, was less devastating to their identity, or because they are from more supportive backgrounds [8].

## Towards a Model of Addiction that Supports Recovery

People struggling with addiction are neither powerless over their addiction, nor are they fully in control [33]. I argued that what would help their recovery best is a model that acknowledges both their agential capacities, as well as the challenges imposed on their agency. But how can we do this within the current dichotomy of choice-or-disease? We are almost forced to choose sides and to either focus on loss of control in addiction, or claim that addicted people have agency they can use. How can we fairly acknowledge both addicted people’s experiences of powerlessness and their experiences of agency?

When I look closer at the life stories of the addicted persons I interviewed, two things stood out. Throughout their using careers, they showed many instances of agency and control over their substance use. Many people were addicted for years, and in these years they showed a variety of control over their use. Some people would use substances recreationally for years before they became addicted. Others plunged into a desperate addiction straight away, but would, over the years, find ways in which they could use with more control. For instance, they would do this by changing their substance of choice, or setting personal rules for themselves. Others would control their use for several weeks, months, or years, and then fall into a binge for a similar period of time. There was not a simple answer to whether or not people had control. They had control to a certain extent, or in a certain situation, or in a certain phase of their lives. But many people described one or several periods in their lives in which they were totally out of control, in which their behaviour utterly bewildered them. I would like to call this the disease *stage* of addiction. But this disease stage was just a stage, surrounded by stages in which people had full or partial control over their use.

So although addiction is not a disease, there can be a disease stage in addiction. To call it a disease *stage* emphasises that this condition may pass, that it is not carved in stone. However, it also acknowledges that there is a stage in addiction in which it is utterly hard to exercise self-control. In this formulation ‘disease’ refers to addictive behaviour being largely impervious both to the agent’s values and to available techniques of self-control [34]. A disease stage does not indicate that agency is completely lost, but that it is very much harder to exercise agency at that stage.

There are several reasons to call this stage a *disease* stage, however, I will also propose an alternative that might be more conceptually correct. The word ‘disease’ reflects the intuitions of most addicted and lay people: that it is a condition someone does not want, and that this condition is harmful. Using the words ‘disease stage’ shows a compromise to the disease model, rather than rejecting the model all together. However, the word ‘disease’ is associated with a physiological cause of the condition. In so far as neurobiological changes cause the automatic addictive behaviour, the word ‘disease’ might be right. However, as Lewis and many others have argued, there are many other causes of addiction: psychological, environmental, and so on.

Although the disease stage is characterised by neuroscientific mechanisms becoming dysfunctional, other issues could have preceded this strong physical dependency: a developmental issue, a sociological issue, or a genetic vulnerability. Although the addicted behaviour at that moment is strongly motivated by a neurological dysfunction, this does not mean that the only treatment lies in the neurological realm. In this sense it can be misleading to still use the word *disease*.

So what are the alternatives to the label ‘disease stage’? In his book Lewis briefly refers to a compulsive stage of addiction. However, compulsion might be too strong because it is associated with a total lack of agency. Lewis defines addiction as habitual behaviour, however, to speak of ‘a habitual stage’ might be too weak, since, as Lewis argues, it is a very relentless, destructive, layered habit. Pickard once suggested that although addiction is a choice, it is often a choice made under duress [35]. Watson has made a similar plea [36]. He defines duress as the agent being ‘subject to coercive circumstances sufficient to compromise her capacity for self-control. It shouldn’t matter whether these circumstances result from human design or natural forces’ (p. 613). He distinguishes this from legal duress: ‘To be addicted (on our assumptions) is to be disposed, perhaps episodically, to volitional impairment under certain circumstances, whereas in the standard case, the duress is a one-shot deal’ (p. 615). Watson proposes, inspired by Morse, a plea of ‘guilty but partially responsible’, Pickard argues for a concept of ‘responsibility without blame’. These concepts simultaneously emphasise that people have agency and that their agency is compromised.

Using the word ‘duress’ would also have my preference. Instead of calling addiction a disease, we can state that addiction has a duress stage, in which the addictive behaviour is largely impervious to the agent’s values and to available techniques of self-control. However, the agent can overcome this stage by developing new techniques of self-control, by building on their self-concept and belief in self-efficacy, by changing their environments and habits, and by engaging in projects that are meaningful to them. As one of the respondents stated:

I think it is definitely a disease but it can become a choice. You can choose to not have the disease, do you know what I mean? (…) You can get to a point where you can choose not to be diseased anymore but it definitely is a disease. It’s like a really bad flu that you know you have to fight off. (…) You can choose to fight it off just by becoming healthier and making smarter decisions. Like instead of going out in the wet and rain without a raincoat you wouldn’t do that to get sick, (chuckling), just doing smarter things, yeah. (R40C)

In this duress state of addiction people need elaborate acts of personal agency to overcome their addiction. They need to believe in their self-efficacy, but they need to develop multiple strategies as well. They probably need several attempts, and some outside support, before they master it.

## Conclusion

Lewis’s work offers us a very much to be welcomed neurobiology of recovery. It offers addicted people a hopeful and respectful narrative for their recovery that treats them as agents rather than as damaged brains. However, his account would have been stronger if he would not have been seduced to take a position in the disease dichotomy. He now risks emphasising people’s own choice and narrative agency too much.

I argued that restoring people’s sense of self-efficacy needs more than just telling that they can do it because they do not have a disease. They need to experience success as well in their attempts. They need to be realistic about the hardships they will encounter, and how they can overcome them. I argued that the narrative recovery might be hard to achieve for some people.

I propose replacing the notions of addiction as a disease or addiction at a choice with the notion that some addictions know a duress-like stage. This is a stage at which it is hard for people to exercise their self-control, but during which their agency is only temporarily compromised. It is also a stage that can be overcome, step by step, and probably after several attempts.

## Footnotes

1 Addiction is a habit that becomes compulsive [1, p.33].

2 An exception to this might be Alexander’s rat park study, although one can argue whether this is a classical animal study.

3 Lewis’s book is a nice response to this impasse: he tries to describe a neuroscience of recovery from addiction, instead of a neuroscience of impairment.

4 the ego-depletion hypothesis is currently highly controversial following the finding of a multi-lab preregistered trial that found that the effect size was at best very small; [37]; see also [38].

5 Rather than trying to restore our synchronic self-control directly, we can restore it indirectly by restoring our diachronic self-control.

6 Although one can also turn this around and argue that people who fail to recover are more likely to support the disease model.

7 For details on the study methodology, see [31].
